# Optimal Message Bundling with Delay and Synchronization Constraints in Wireless Sensor Networks

**DOI:** 10.3390/s19184027

**Published:** 2019-09-18

**Authors:** Xintao Huan, Kyeong Soo Kim, Sanghyuk Lee, Moon Keun Kim

**Affiliations:** 1 Department of Electrical and Electronic Engineering, Xi’an Jiaotong-Liverpool University (XJTLU), Suzhou 215123, China; Xintao.Huan@xjtlu.edu.cn (X.H.); Sanghyuk.Lee@xjtlu.edu.cn (S.L.); 2 Department of Architecture, Xi’an Jiaotong-Liverpool University (XJTLU), Suzhou 215123, China; Moon.Kim@xjtlu.edu.cn

**Keywords:** energy efficiency, message bundling, end-to-end delay, time synchronization accuracy, wireless sensor networks

## Abstract

Energy efficiency and end-to-end delay are two of the major requirements for the monitoring and detection applications based on resource-constrained wireless sensor networks (WSNs). As new advanced technologies for accurate monitoring and detection—such as device-free wireless sensing schemes for human activity and gesture recognition—have been developed, time synchronization accuracy becomes an important requirement for those WSN applications too. Message bundling is considered one of the effective methods to reduce the energy consumption for message transmissions in WSNs, but bundling more messages increases the transmission interval of bundled messages and thereby their end-to-end delays; the end-to-end delays need to be maintained within a certain value for time-sensitive applications like factory monitoring and disaster prevention, while the message transmission interval affects time synchronization accuracy when the bundling includes synchronization messages as well. Taking as an example a novel WSN time synchronization scheme recently proposed for energy efficiency, we investigate an optimal approach for message bundling to reduce the number of message transmissions while maintaining the user-defined requirements on end-to-end delay and time synchronization accuracy. Formulating the optimal message bundling problem as integer linear programming, we compute a set of optimal bundling numbers for the sensor nodes to constrain their link-level delays, thereby achieving and maintaining the required end-to-end delay and synchronization accuracy. Extensive experimental results based on a real WSN testbed using TelosB sensor nodes demonstrate that the proposed optimal bundling could reduce the number of message transmissions about 70% while simultaneously maintaining the required end-to-end delay and time synchronization accuracy.

## 1. Introduction

In a typical wireless sensor network (WSN) based on lots of battery-powered sensor nodes, minimizing the energy consumptions at the sensor nodes is critical to the service time of the WSN. Considering that the radio activities consume the most energy at sensor nodes, reducing the number of message transmissions is key to saving the energy [1,2], and the data bundling (The terms of “data bundling” and “message bundling” are used interchangeably in this paper.). is an efficient way to achieve it [3,4,5]. Depending on the operation locations, the existing data bundling schemes (e.g., [4,5,6,7,8,9,10,11,12]) can be classified into in-node, in-network, and hybrid bundling. However, one major disadvantage of employing data bundling schemes is the increase of end-to-end (E2E) delay, which draws much attention in WSN research [13,14,15,16,17]. By E2E delay, we mean the difference between the measurement time at an originating sensor node and the reception time of the resulting measurement data by the head node via a message. The data bundling has been studied so far in the context of energy efficiency and E2E delay [4,5,11,12], but synchronization accuracy is hardly considered as one of the requirements because conventional energy-efficient time synchronization schemes are mostly designed independent of data bundling.

Note that, for most long-term monitoring and detection applications in WSNs, energy-efficient and high-precision time synchronization is crucial to ordering and analyzing the data measured over a long period of time. For instance, in human activity and gesture recognition based on the novel device-free wireless sensing [18,19], the sensed data must be ordered accurately and processed timely, which requires high-precision time synchronization for the former while demands certain latency—i.e., E2E delay—for the latter. Among many research works on WSN time synchronization aiming at improving time synchronization accuracy while lowering energy consumption [1,20,21,22,23,24,25,26], the energy-efficient time synchronization scheme we proposed in [1], which we call it EE-ASCFR—i.e., short for *E*nergy-*E*fficient time synchronization scheme based on *A*synchronous *S*ource *C*lock *F*requency *R*ecovery—throughout the paper—leverages data bundling to further reduce the energy consumption in terms of the number of synchronization message transmissions; thanks to the reverse two-way message exchange scheme adopted in EE-ASCFR, the time synchronization is done at the head node and the corresponding synchronization data are bundled together with measurements into a bundled message at a sensor node. Consequently, the time synchronization accuracy is tied to the interval of the bundled message transmissions from the sensor node to the head node, i.e., the synchronization interval (SI). This means that the number of measurement data in a bundled message is linked to the interval of the bundled message transmissions and thereby not only affects the aforementioned E2E delay, but also the time synchronization accuracy. In such a case, we need to optimize the number of bundled messages under the constraint of E2E delay and time synchronization accuracy in achieving higher energy efficiency.

In this paper, we formulate the aforementioned bundling optimization problem as integer linear programming (ILP), where the number of bundled messages for each sensor node is optimized while jointly satisfying the user-defined performance requirements on E2E delay and time synchronization accuracy. In this way, we can further reduce the energy consumption through data bundling while meeting the requirements.

The rest of this paper is organized as follows: Section 2 provides the preliminaries of this study where we introduce the time synchronization scheme based on the data bundling procedure and discuss the related conflicts of performance metrics. Section 3 presents our proposed approach for optimal message bundling with delay and synchronization constraints and its formulation as ILP. Section 4 exhibits our system design at both head (The head node and the monitoring station—e.g., PC or server connected to it—are jointly called head throughout this paper.) and sensor node. Section 5 demonstrates the performance of the proposed approach through experimental results on a real WSN testbed. Section 6 concludes our work in this paper and discusses future works.

## 2. Preliminaries

Most WSNs have the fundamental characteristics of limited resources, multi-hop communication, large scale and dynamic environments [27]. To achieve satisfactory performance in typical WSN applications including environmental monitoring, event detection, and industrial applications, several specific requirements—e.g., E2E delay and synchronization accuracy—have to be met. With consideration of the most basic requirements of energy efficiency, jointly maintaining the three metrics is crucial; however, it has not been addressed in the existing research.

### 2.1. Existing Methods toward Improving Energy Efficiency

In the literature, diverse methods across different layers have been proposed to improve the energy efficiency: transmission power control and multi-channel communication in the physical layer [28,29,30,31,32,33]; maximum (re-)transmission times and retry delay tuning in the media access control (MAC) layer [17,34,35]; packet payload size and sampling rate adaptation in the application layer [36,37,38], as well as cross-layer methods such as [39].

The data bundling method employed in this paper belongs to the category of packet payload size adaptation since tuning the bundling number directly affects the packet payload size. Note that data bundling is a mature and effective method that has been leveraged in both the conventional optical burst switching [40] and the novel mobile wireless network [41], the latter of which adopts it to minimize the memory and communication usage, thereby the energy efficiency is improved. Note that one notable difference of the data bundling method in this paper compared to those in other areas is that it is investigated as an optimal bundling problem under the constraint of time synchronization accuracy in addition to the E2E delay one.

### 2.2. Energy-Efficient Time Synchronization Schemes Using Data Bundling

**Figure 1:**
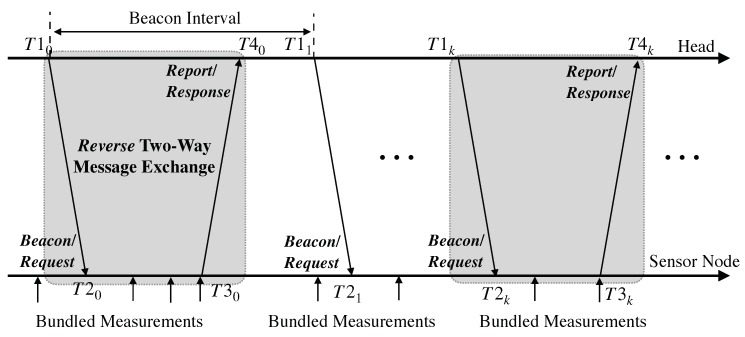
The reverse two-way message exchange and data bundling illustrated in EE-ASCFR [1].

To fulfill the desired requirements of energy efficiency and synchronization accuracy, many schemes have been proposed such as [1,20,21,22,23,24,25,26]. Among those, EE-ASCFR proposed in [1] particularly suits the E2E delay calculation since computing the E2E delay at the head is completely in conformity with the preferential asymmetric scenario of EE-ASCFR. Two approaches of reverse two-way message exchange and data bundling are leveraged to reduce the message transmissions as demonstrated in Figure 1; the latter data bundling is the major contributor for conserving energy. We will discuss this in detail later in this section. Nevertheless, the time synchronization is accomplished through the former reverse two-way message exchange, in which the first-order affine clock model is used to model the hardware clock $T_{i}$ of a sensor node *i* with respect to the reference clock *t* of the head node. Since the hardware clocks of the sensor nodes have different clock frequency ratios and clock offsets with respect to the reference clock due to their coarse crystal oscillators, the following equation is employed in EE-ASCFR to represent the hardware clock of the sensor node *i*: for $i\;\in \;\left[0,1,\ldots ,N-1\right]$,

(1) $$T_{i}\left(t\right)=\left(1+\epsilon_{i}\right)t+\theta_{i},$$

where $\left(1+\epsilon_{i}\right)\in R_{+}$ and $\theta_{i}\in R$ represent the clock frequency ratio and the clock offset, respectively [1]. The corresponding logical clock $T_{i}$ can be described based on Equation (1) as follows: For $t_{k}\;<\;t\;\leq \;t_{k+1}$ (k=0,1,…),

(2) $$T_{i}\left(T_{i}\left(t\right)\right)=T_{i}\left(T_{i}\left(t_{k}\right)\right)+\frac{T_{i}\left(t\right)-T_{i}\left(t_{k}\right)}{1+{\hat{\epsilon }}_{i,k}}-{\hat{\theta }}_{i,k},$$

where the reference time for the *k*th synchronization is denoted by $t_{k}$, the estimated clock skew and offset from the *k*th synchronization are described as ${\hat{\epsilon }}_{i,k}$ and ${\hat{\theta }}_{i,k}$. Note that, as mentioned in [1], ${\hat{\theta }}_{i,k}$ is set to 0 in Equation (2) since its compensation is done at the head node; in addition, the asynchronous SCFR scheme introduced in [42] is employed for the sensor node to synchronize its frequency of the logical clock to that of the reference clock. Furthermore, asymmetric high-precision time synchronization (AHTS) [43] is proposed as an improvement of EE-ASCFR, which is later leveraged in our proposed optimal bundling, to address the practical application and multi-hop scenario.

However, because the synchronization accuracy (SA) is affected by SI as illustrated in Table 1 (i.e., simulation results of EE-ASCFR and practical evaluation results of AHTS), we need to maintain SI to a reasonable threshold for better synchronization accuracy. Based on those evaluation results in Table 1, the relationship (R) between SI and synchronization accuracy is represented as follows:

(3) SI=R(SA),

in which the requirement of SA could be translated to the requirement of SI.

**Figure 2:**
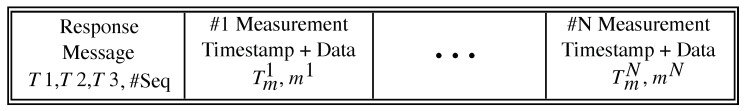
Payload contents of the data bundling procedure introduced in EE-ASCFR [1].

**Figure 3:**
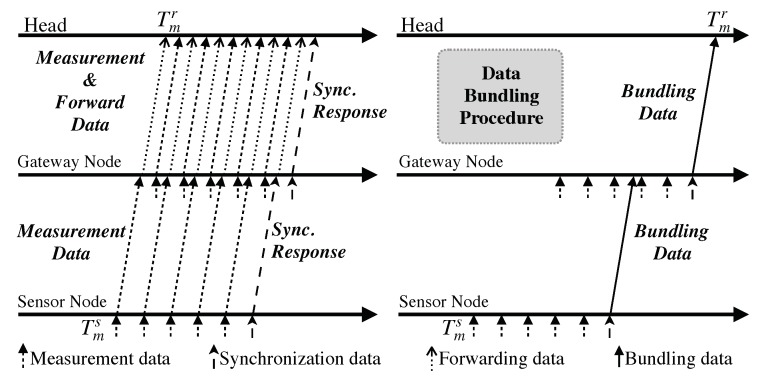
Comparison of the message transmissions of the time synchronization schemes with and without data bundling procedure.

The energy consumption caused by message transmissions could be drastically reduced by the aforementioned data bundling procedure as demonstrated in Figure 1: downstream, the synchronization “Request” message for timestamp T1 is embedded inside a regular beacon message; upstream, the synchronization “Response” message for timestamps T1, T2, and T3 is bundled together with measurement data as shown in Figure 2. Note that bundling more measurement data could lead to better energy efficiency, which, however, increases SI and decreases SA as discovered in the evaluation of [1] and [43]. Nevertheless, as previously mentioned, EE-ASCFR only covers the single-hop scenario, the more asymmetric approach (i.e., AHTS), which is based on EE-ASCFR and extends it to multi-hop resource-constrained sensor networks, is employed in the proposed approach to cover multi-hop scenarios. The data bundling procedure is inherited and extended to the multi-hop case as illustrated in Figure 3. Of particular note is that the bundling number is not discussed either in EE-ASCFR or AHTS since it directly affects the E2E delay and synchronization accuracy, which makes its value selection very complicated.

### 2.3. Conflicts of Performance Metrics: Energy Efficiency, E2E Delay and Synchronization Accuracy

Simultaneously meeting the requirements for the three performance metrics of energy efficiency, E2E delay, and synchronization accuracy is not possible due to their relationship discussed in Section 2.2. Time synchronization provides the possibility of accurate calculation and maintenance of E2E delay, however, employing the time synchronization scheme requires certain computing and power resources, which produces the conflict between energy efficiency and E2E delay calculation. Although the data bundling procedure introduced in EE-ASCFR could drastically reduce the energy consumption for message transmissions and bundling more data could lead to higher energy efficiency, the data bundling procedure could directly result in high E2E delay as revealed in Figure 3, which is the conflict between energy efficiency and E2E delay maintenance. Specific to the delay calculation illustrated in Figure 3, the E2E delay—i.e., $T_{m}^{r}$−$T_{m}^{s}$—of measurement *m*, would be relatively small (e.g., multiples of forwarding delay which typically in milliseconds) in the regular direct forwarding method. However, due to the bundling procedure in the intermediate sensor nodes, the E2E delay could be as large as multiples of measurement interval. Nonetheless, the bundling procedure is shown to be quite efficient in conserving energy by reducing the number of message transmissions as shown in Figure 3, which is critical to low-power sensor nodes. As for synchronization accuracy, SI should be shorter for achieving higher accuracy [1], but shorter SI results in more message transmissions, which again leads to higher energy consumption. This draws forth the conflict between energy efficiency and synchronization accuracy.

To jointly meet the three performance requirements, comprehensive optimizations should be taken so that overall satisfactory performances among those three requirements could be achieved. In this paper, a new approach for the bundling number optimization for each sensor node is proposed to fulfill the requirement of maintaining E2E delay and synchronization accuracy while minimizing the message transmissions.

## 3. ILP Model for the Optimal Bundling Problem

In a WSN with *N* sensor nodes, the energy consumption $e_{i}^{t}$ for the message transmissions at sensor node *i* is modeled as follows: For $i\in \left[0,1,\ldots ,N-1\right]$,

(4) $$e_{i}^{t}=\alpha_{i}e_{i}^{m}+\beta_{i}e_{i}^{s}+\gamma_{i}e_{i}^{f},$$

where $e_{i}^{m}$ and $e_{i}^{s}$ denote the energy consumption for the transmission of a measurement and a synchronization message generated by sensor node *i*, respectively, and $e_{i}^{f}$ is the energy consumption for forwarding either a synchronization or a measurement message from offspring sensor nodes at sensor node *i*. The coefficients $\alpha_{i}$, $\beta_{i}$ and $\gamma_{i}$ are the number of transmissions for corresponding messages.

If we apply data bundling, Equation (4) can be modified as follows:

(5) $$e_{i}^{t}=\left\{\begin{array}{cc} \delta_{i}e_{i}^{b}, & \mathrm{all}\;\mathrm{data}\;\mathrm{bundling},\\ \delta_{i}e_{i}^{b}+\gamma_{i}e_{i}^{f}, & \mathrm{self}\;\mathrm{data}\;\mathrm{bundling}, \end{array}\right.$$

where $\delta_{i}e_{i}^{b}$ is the total energy consumption caused by transmitting the bundled messages. Note that, unlike the all data bundling option, which bundles all data into one bundled message, the self data bundling option bundles only the data generated from the sensor node itself, which is the reason the term $\gamma_{i}e_{i}^{f}$ still remains. From the network-level perspective, the total energy consumption for message transmissions in the network could be described as follows:

(6) $$E^{t}=\sum_{i=0}^{N-1}e_{i}^{t}.$$

### 3.1. Maximization of Bundling Number for Energy Efficiency

With the model for energy consumption caused by message transmissions, we can increase the network energy efficiency by minimizing the total energy consumption for message transmissions (i.e., $E^{t}$).

As shown in Equation (5), a large number of bundled message transmissions (i.e., $\delta_{i}$) could result in more energy consumption, but it could be decreased by bundling more messages in one transmission. Let $\Gamma^{i}$ be the number of bundled messages at sensor node *i*. Then, a larger $\Gamma^{i}$ could lead to a smaller $\delta_{i}$, which could reduce total energy consumption for message transmissions. Consequently, we can minimize $E^{t}$ by maximizing $\Gamma^{i}$.

Even though we can increase the amount of bundling for better energy efficiency, we cannot indefinitely because the E2E delay of the measurement data are sensitive to the number of bundling. Therefore, the maximization of bundling number for energy efficiency can be formulated as follows:

(7) $$\begin{array}{cc} \; & \mathrm{maximize}\;\Gamma \\ \; & \mathrm{subject}\;\mathrm{to}\;\chi^{min}\leq \Gamma^{i}\leq \chi^{max},\forall i\in \left[0,\ldots ,N-1\right], \end{array}$$

where

$$\Gamma =\sum_{i=0}^{N-1}\Gamma^{i}.$$

Note that Γ is the total bundling number in the network and that $\chi^{min}$ and $\chi^{max}$ are the lower and upper bounds of the measurement bundling number which are application-specific parameters and could be specified by the user.

### 3.2. Constraining E2E Delay

We define the E2E delay of sensor node *i* as the difference between the time of a certain measurement *m* at the sensor node and the time of the reception of the resulting message by the head:

(8) $$D_{e2e}^{i}≜\sum_{l=0}^{L-1}D_{l}^{i}=T_{m}^{i,r}-T_{m}^{i,s},$$

where $D_{l}^{i}$ is the link delay at link *l* of *L* links from sensor node *i* to the head, $T_{m}^{i,r}$ and $T_{m}^{i,s}$ are the receiving time at the head and the measuring time at the sensor node respectively, the latter of which is a time with respect to the reference clock at the head translated by a time synchronization scheme. Specifically, the $D_{e2e}^{i}$ is a path-level delay which consists of several link-level delays in the network. Considering the bundling procedure, the link delay at link *l* for sensor node *i* could be described as follows:

(9) $$D_{l}^{i}=D_{prop}^{i,l}+D_{serv}^{i,l}+D_{bund}^{i,l},$$

where $D_{prop}^{i,l}$ denotes the propagation delay, which is typically at the nanosecond level in WSN, $D_{bund}^{i,l}$ is the delay caused by the bundling procedure which is in multiples of the measurement interval (e.g., 5× 1 s for the measurement interval of 1 s and the bundling number of 5). Based on the service time model [39] for TinyOS, which is running on the TelosB sensor node, we can model $D_{serv}^{i,l}$ as follows:

(10) $$D_{serv}^{i,l}=\left\{\begin{array}{cc} D_{SPI}^{i,l}+D_{succ}^{i,l}+\left(N_{try}^{i,l}-1\right)\;\cdot \;D_{retry}^{i,l}, & N_{try}^{i,l}\leq N_{max}^{i,l},\\ D_{SPI}^{i,l}+D_{fail}^{i,l}+\left(N_{max}^{i,l}-1\right)\;\cdot \;D_{retry}^{i,l}, & N_{try}^{i,l}>N_{max}^{i,l}, \end{array}\right.$$

where

$$\begin{array}{cc} D_{succ}^{i,l} & =D_{MAC}^{i,l}+D_{frame}^{i,l}+D_{ACK}^{i,l},\\ D_{fail}^{i,l} & =D_{MAC}^{i,l}+D_{frame}^{i,l}+D_{waitACK}^{i,l},\\ D_{retry}^{i,l} & =T_{retry}^{i,l}+D_{frame}^{i,l}+D_{waitACK}^{i,l}. \end{array}$$

Note that the delay parameters—i.e., one-time serial-peripheral interface (SPI) bus loading delay $D_{spi}^{i,l}$, medium access control (MAC) layer delay $D_{MAC}^{i,l}$, frame transmission delay $D_{frame}^{i,l}$, acknowledgment (ACK) transmission delay $D_{ACK}^{i,l}$ and ACK waiting delay $D_{waitACK}^{i,l}$—in the above equations are platform-dependent values, and their values are typically in the order of milliseconds. In addition, $N_{try}^{i,l}$ and $N_{max}^{i,l}$ are the current and the maximum allowed number of transmissions for a successful delivery, and $T_{retry}^{i,l}$ is the user-defined backup time of retransmission. For simplicity and energy efficiency, the packet retransmission is not taken into account in our proposed scheme since there are usually not many packet retransmissions in the network with lower traffic. Thus, Equation (10) could be simplified as follows:

(11) $$D_{serv}^{i,l}=D_{SPI}^{i,l}+D_{MAC}^{i,l}+D_{frame}^{i,l}+D_{ACK}^{i,l},$$

where the value of $D_{serv}^{i,l}$ is around 10 based on the reference values in [39].

The link delay in Equation (9) could be further simplified when the measurement interval for an application ($I_{meas}^{i,l}$) is much larger than the service delay ($D_{serv}^{i,l}$): because $D_{bund}^{i,l}>I_{meas}^{i,l}$, $I_{meas}^{i,l}\gg D_{serv}^{i,l}$ also implies $D_{bund}^{i,l}\gg D_{serv}^{i,l}$. Hence,

(12) $$D_{link}^{i,l}\approx D_{bund}^{i,l},\;\mathrm{if}\;I_{meas}^{i,l}\gg D_{serv}^{i,l}.$$

Note that the service delay $D_{serv}^{i,l}$ should not be ignored in case the application requires frequent measurements, i.e., $I_{meas}^{i,l}$ is comparable to $D_{serv}^{i,l}$.

In typical hierarchical multi-hop WSNs, sensor nodes located in different layers handle different amounts of traffic: for instance, the gateway node in the upper layer has to handle the message traffic from its offspring sensor nodes as well as itself; the higher layer it is located in, the more message traffic it has to handle. This means that even two sensor nodes with the same bundling number (i.e., $\Gamma^{i}$) could have different bundling delays due to the variance in their message traffic. By introducing a message traffic coefficient ($\frac{1}{1+\lambda^{i}}$) for each sensor node that periodically measures data with the same measurement interval (i.e., $I_{meas}^{i}$), the bundling delay at sensor node *i* ($D_{bund}^{i}$) could be represented as follows:

(13) $$D_{bund}^{i}=\frac{\Gamma^{i}}{1+\lambda^{i}}\;\cdot \;I_{meas}^{i},$$

where $\lambda^{i}$ denotes the number of offspring sensor nodes. Then, the $D_{e2e}^{i}$ for the applications with normal measurement interval could be modeled as follows:

(14) $$D_{e2e}^{i}=\sum_{l=0}^{L-1}D_{bund}^{i}.$$

With Equation (14), we can also constrain the E2E delay in the optimal bundling problem in Equation (7) with the user-defined E2E delay requirement ($D_{e2e}^{max}$): for i∈[0,1,…,N−1],

(15) $$D_{e2e}^{i}\leq D_{e2e}^{max}.$$

### 3.3. Constraining Synchronization Accuracy

Since the proposed optimal message bundling approach is based on the reverse asymmetric time synchronization scheme, the synchronization accuracy depends on the report interval of the synchronization messages, which, in turn, are carried by the bundled messages. In such a case, the user-required synchronization accuracy (${\mathrm{SA}}^{min}$) could be maintained through constraining the E2E delay of the bundled message when sensor nodes generate measurement data periodically. Based on the empirical sets—i.e., R—of the relationships between synchronization accuracy and SI previously provided in Section 2, the user-required synchronization accuracy could be translated to the delay requirement as follows:

(16) $$D_{e2e}^{SA}=R\left({\mathrm{SA}}^{min}\right).$$

Combining Equations (14) and (16), the synchronization accuracy could be achieved through constraining the E2E delay as follows: For i∈[0,1,…,N−1],

(17) $$D_{e2e}^{i}\leq D_{e2e}^{SA}.$$

### 3.4. ILP Model

Combining the objective function Equation (7) and the two constraint sets Equations (15) and (17), we can formulate the optimal bundling problem as the following ILP:

(18) $$\begin{array}{cc} \; & \mathrm{maximize}\;\Gamma =\sum_{i=0}^{N-1}\Gamma^{i},\\ \; & \mathrm{subject}\;\mathrm{to}\;\\ \; & \begin{array}{cc} \; & \;\;\;\;\chi^{min}\leq \Gamma^{i}\leq \chi^{max},\;\forall i\in \left[0,\ldots ,N-1\right],\\ \; & \;\;\;\;D_{e2e}^{i}\leq \min\left(D_{e2e}^{max},R\left({\mathrm{SA}}^{min}\right)\right),\;\forall i\in \left[0,\ldots ,N-1\right], \end{array} \end{array}$$

where the E2E delays of all sensor nodes are jointly constrained by the user-defined E2E delay $D_{e2e}^{max}$ and the synchronization accuracy ${\mathrm{SA}}^{min}$ requirements, and the bundling number of each sensor node is constrained by the user-defined lower $\chi^{min}$ and upper $\chi^{max}$ bounds, respectively. Applying the set of optimal bundling numbers computed from this ILP model to the sensor nodes, the required E2E delay and synchronization accuracy could be jointly maintained while the bundled message transmissions are still minimized.

## 4. System Design

**Figure 4:**
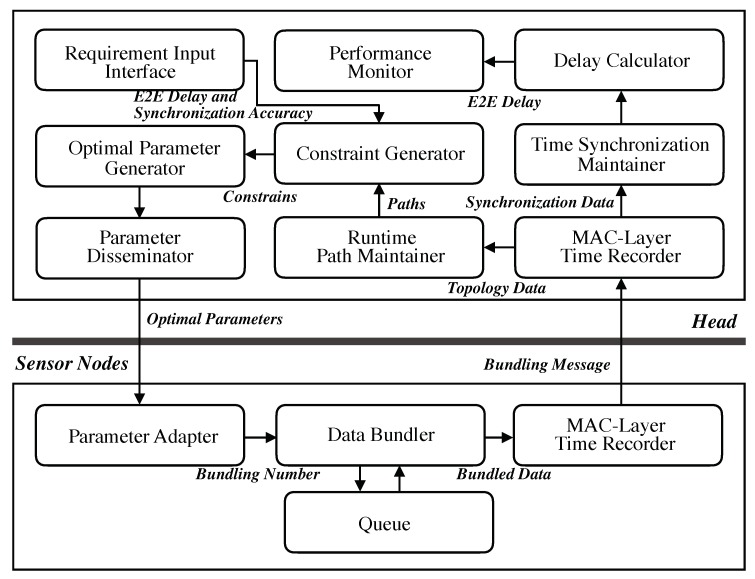
System architecture of the proposed optimal bundling.

Figure 4 shows a system architecture for the proposed optimal bundling based on the ILP model formulated in Section 3.4, where the two subsystems—i.e., the *Performance Maintainer* at the head and the *Parameter Adapter* at each sensor node—are built to achieve the optimization target. This separation of components further releases the computational burden of the sensor node: the head is in charge of monitoring the runtime topology and computing the optimal bundling number for each sensor node dynamically; the sensor node, therefore, is relieved from the complex computation and leverages the optimal result from the head straightforwardly.

### 4.1. Performance Maintainer at Head

Since the time synchronization operation is centralized at the head in EE-ASCFR and AHTS, we first build the reference time synchronization system at the head as a component of the proposed system. The time synchronization is achieved through the *MAC-Layer Time Recorder* and the *Time Synchronization Maintainer*, the latter of which translates timestamps between the head and a sensor node. With the time synchronization component, a measurement timestamp recorded at the sensor node—i.e., $T_{m}^{s}$ in Figure 3—is translated to a timestamp based on the hardware clock of the head. Then, the E2E delay is obtained as a difference between the translated measurement timestamp and the receiving timestamp of $T_{m}^{r}$ through the *Delay Calculator*. Afterwards, the runtime E2E delay is monitored through the *Performance Monitor*.

Based on the topology data carried in each bundled message, the routing paths for all sensor nodes are recovered in the *Runtime Path Maintainer*, and one set of paths is generated and delivered to the *Constraint Generator*. The user-defined requirements of E2E delay and synchronization accuracy are captured through the user interface of *Requirement Input Interface*. By combining the performance requirements and the current path information, the *Constraint Generator* generates a set of constraints and passes it to the *Optimal Parameter Generator*, where the optimal bundling number for each sensor node is obtained as a solution of the ILP model. Finally, the optimal bundling numbers from the *Optimal Parameter Generator* are delivered to sensor nodes by the *Parameter Disseminator*.

Of particular note is that, whenever the topology is updated or the users change their requirements dynamically, the whole process above will be operated to provide up-to-date optimal bundling numbers. In addition, since the proposed system is in a centralized manner, the major trade-off is the communication overhead for disseminating the optimal results to the sensor nodes. This overhead, by the way, would not be a major issue in practice unless topology updates and user requirements changes are too frequent.

### 4.2. Parameter Adapter at Sensor Nodes

To reduce the computational complexity required by the proposed optimal bundling on the sensor nodes, three lightweight components (including the *MAC-Layer Time Recorder* from the time synchronization scheme) are implemented at sensor nodes. The *Parameter Adapter* receives the optimal bundling number and delivers it to the *Data Bundler* which bundles that number of measurement data temporarily stored in the *Queue* plus timestamps into one message. Then, the bundled message will be again timestamped for T3 by the *MAC-Layer Time Recorder* as shown in Figure 1 and Figure 4.

## 5. Experimental Results

**Figure 5:**
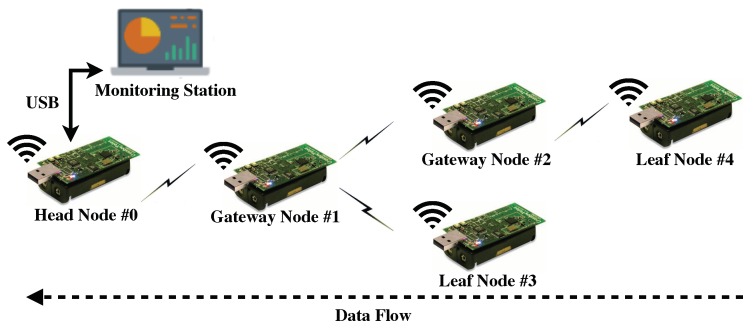
Experiment setup of a real three-hop wireless sensor network (WSN) testbed consisting of five TelosB sensor nodes.

**Figure 6:**
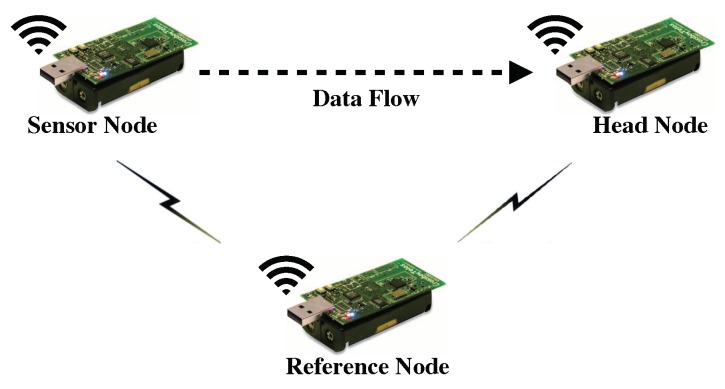
Evaluation of time synchronization accuracy using one additional reference node.

The proposed approach is implemented on a real three-hop WSN testbed consisting of five TelosB sensor nodes as illustrated in Figure 5. To verify the time synchronization accuracy of the time synchronization approach embedded in the proposed optimal bundling, we establish the time synchronization accuracy measurement based on the setup illustrated in Figure 6. Similar to the approach of Reference-Broadcast Synchronization (RBS) [44], one reference node is employed to broadcast the reference message to the head and sensor node under evaluation to record the receiving timestamps. With neglecting the propagation delay, those receiving timestamps should be at the same time point, thanks to the leveraging of the MAC-layer timestamping [43]. Afterwards, the recorded timestamps in the sensor node under evaluation will be sent to the head for translation. Since the head and sensor node under evaluation are synchronized through the time synchronization approach, so the synchronization error could be computed in the head through comparing the differences of the head’s recorded timestamp and the translated one.

**Figure 7:**
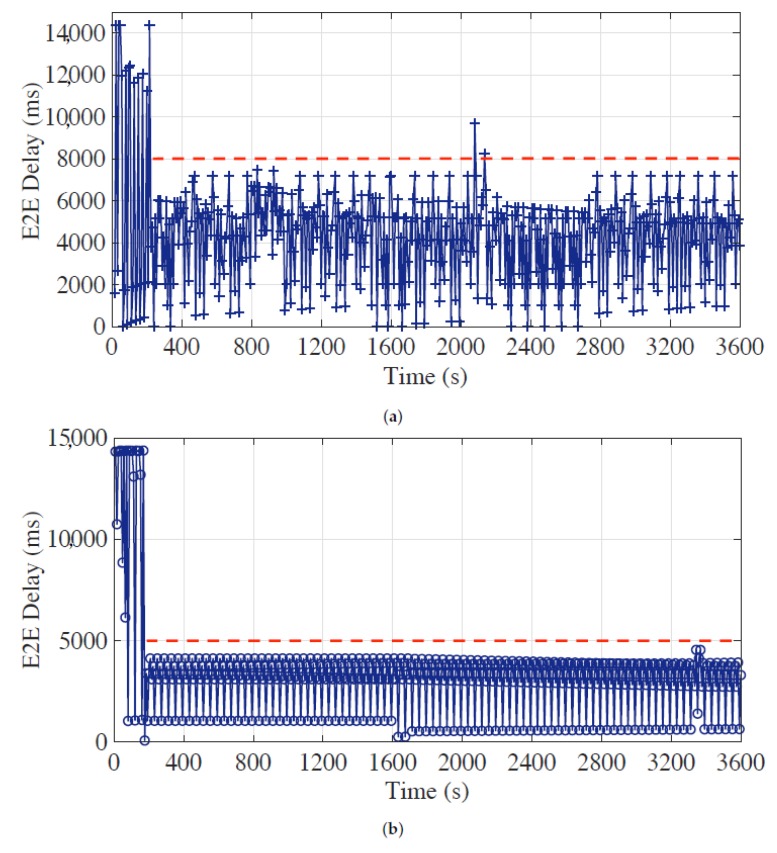
End-to-end (E2E) delay performance of the optimal bundling under static E2E delay requirement setting of (**a**) 8 s and (**b**) 5 s with the maximum bundling number of 15.

**Figure 8:**
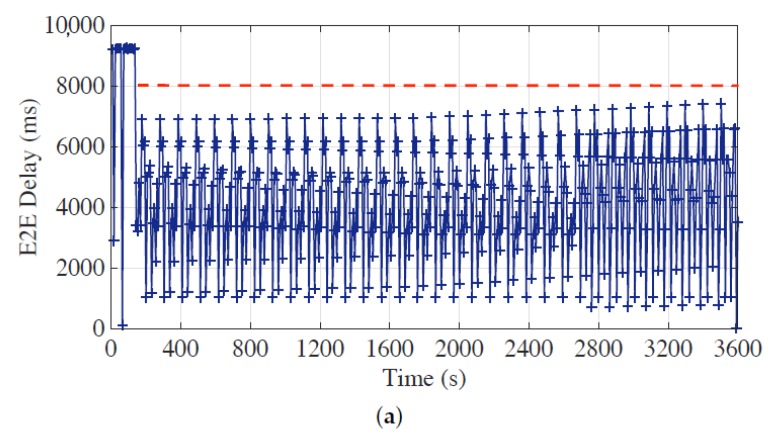

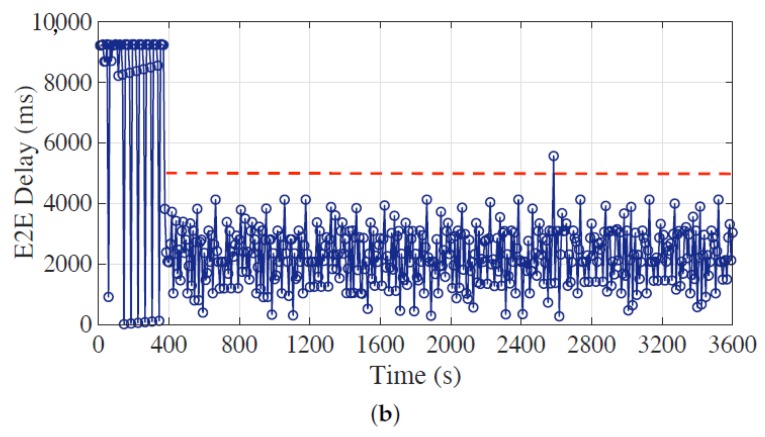
End-to-end (E2E) delay performance of the optimal bundling under static E2E delay requirement setting of (**a**) 8 s and (**b**) 5 s with the maximum bundling number of 10.

**Figure 9:**
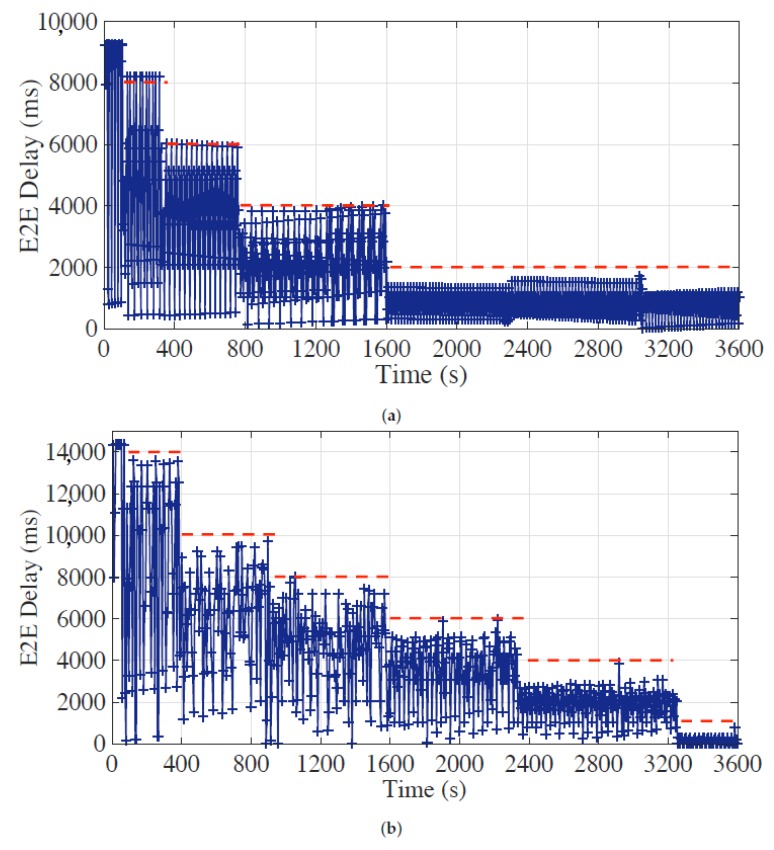
End-to-end (E2E) delay performance of the optimal bundling under dynamic E2E delay requirement setting and the maximum bundling number of (**a**) 10 and (**b**) 15.

During the experiments, each sensor node generates one measurement per second, and the E2E delays of the latest measurements in bundled messages from all sensor nodes are collected and stored in a time sequence in order of their arrivals at the head, which are shown in Figure 7, Figure 8 and Figure 9. The red horizontal dotted lines indicate the E2E delay requirements for corresponding time periods, and the requirement of synchronization accuracy is set to 5 for all experiments.

### 5.1. Delay Performance under Static Requirement Setting

We first evaluate the E2E delay performance of the optimal bundling under static requirement setting—e.g., E2E delay requirement of 8 s and 5 s—with different maximum bundling numbers—i.e., $\chi^{max}$ in Equation (18)—of 15 and 10. We run the experiment for 3600 s to demonstrate the *long-term maintenance capability*.

The performance of the proposed approach under the static requirement setting is explicitly exhibited through diverse experiments with different parameters and requirements. As shown in Figure 7a,b, the E2E delays can exceed 14 s before the optimal bundling is applied. Once the optimal bundling is applied with the requirement of 8 s and 5 s; however, we can see that the E2E delay is controlled and kept under the requirements for most of the time. Note that there are few data points crossed the requirement line; because the gateway node serves its own measurement data first, the data from its offspring nodes, sometimes, could be buffered in the queue and sent by the next available message, which would increase the E2E delay of the corresponding message. Next, we change the maximum bundling number from 15 to 10 to evaluate the proposed optimal bundling approach since the maximum bundling number is often limited by the measurement data length and the maximum payload size of the underlying protocol. Figure 8a,b illustrate that the E2E delay requirement can be well fulfilled with applying the proposed optimal bundling approach.

### 5.2. Delay Performance under Dynamic Requirement Setting

We also evaluate the E2E delay performance of the optimal bundling under dynamic requirement setting with the maximum bundling number of 10 and 15 to demonstrate its *run-time maintenance capability*.

During the evaluation with maximum bundling number of 10, the E2E delay requirement is dynamically changed from 8 s to 2 s step-by-step as shown in Figure 9a. In addition, for the evaluation with maximum bundling number of 15 shown in Figure 9b, we extend the range of E2E delay requirement to 14 s to 1 s to further illustrate the performance under the dynamic scenario. The results demonstrate that the proposed optimal bundling nicely handles multiple dynamic requirements of E2E delay throughout the experiments. As discussed in Section 5.1, however, some data points slightly cross the requirement line, which is due to the neglect of the service time (i.e., $D_{serv}^{i,l}$) in Equation (9). When the optimal bundling numbers are too strict, which just fulfills the requirement, the influence of the neglect of the small service time would be notable. Note that, in the extreme case, when the E2E delay is strict to 1 s as shown in Figure 9b, which reaches the measurement interval of our experiment as previously described in Section 5, the proposed optimal bundling could still fulfill the requirement but with assigning no larger optimal bundling number than 1 to all sensor nodes as shown in Table 2. However, when the E2E delay requirement is much stricter, i.e., smaller than the measurement interval, this may lead to an unsatisfactory situation since there is no room for the proposed optimal bundling to play.

### 5.3. Synchronization Accuracy

**Figure 10:**
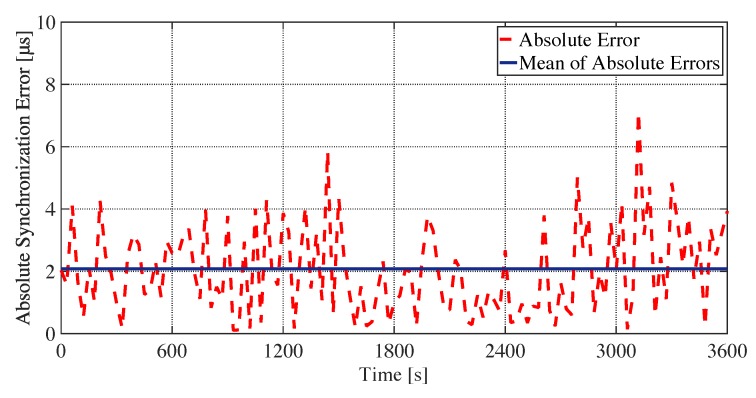
Absolute time synchronization error of node 2 during the experiment of static requirement setting with maximum bundling number of 10 and end-to-end (E2E) delay requirement of 8s illustrated in Figure 8a.

With the novel time synchronization schemes of EE-ASCFR and AHTS, the synchronization accuracy could be fulfilled as far as the E2E delay of the bundled message satisfying the synchronization interval: the synchronization interval—i.e., E2E delay—exhibited in the evaluation results as exhibited in Figure 7, Figure 8 and Figure 9, could overfulfill the SI requirement (e.g., 10 s SI could lead to 2.3385 μs synchronization accuracy as illustrated in Table 1), intuitively the synchronization accuracy could be strictly followed. Furthermore, we take node 2 in the static requirement setting experiment—i.e., experiment shown in Figure 8a with maximum bundling number of 10 and E2E delay requirement of 8 s—as an example, to practically evaluate the time synchronization accuracy. As illustrated in Figure 10, most of the absolute synchronization errors (i.e., the dotted line) are under 5 μs, and the mean of absolute synchronization errors is 2.0849 μs, which could overfulfill the 5 synchronization accuracy requirement.

### 5.4. Energy Efficiency

**Figure 11:**
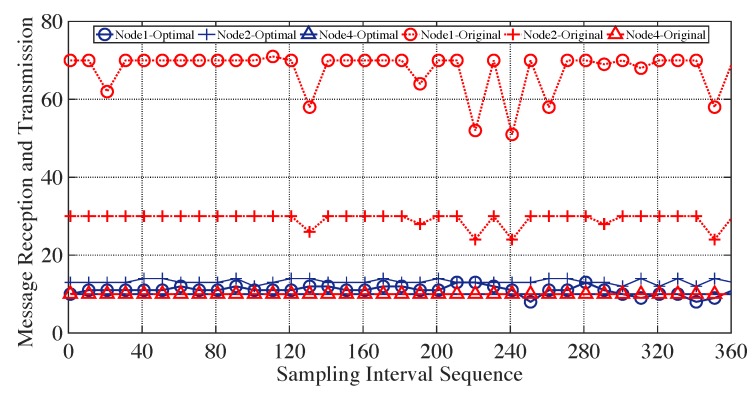
Number of message receptions and transmissions of sensor nodes with and without optimal message bundling for 3600 s with sampling interval of 10 s.

To evaluate the energy efficiency, we indirectly estimate it by comparing the number of message receptions and transmissions with and without optimal bundling. Taking the path of 0↔1↔2↔4 as an example, the number of message receptions and transmissions of the sensor nodes 1,2,4 in the experiment—i.e., experiment exhibited in Figure 7a—of static E2E delay requirement setting is illustrated in Figure 11.

The number of message receptions and transmissions of each sensor node is counted every 10 s over the period of 3600 s. With the proposed optimal bundling, all the sensor nodes could maintain their message receptions and transmissions around the number of 10. Without the optimal bundling, on the other hand, the numbers of their message receptions and transmissions are relatively larger, whose average of 35 is more than triple that of the optimal bundling. In other words, around 70% of message transmissions can be reduced using the proposed optimal bundling in this specific case. Of particular note is that, with the increase of the network size and the maximum bundling number, the performance of the proposed optimal bundling would be even better.

### 5.5. Discussion

As illustrated in Table 2, the optimal bundling numbers that our proposed approach assign to the leaf nodes (i.e., nodes 3 and 4 in our experiment as shown in Figure 5) are relatively small regardless of the variation of the E2E delay requirements. This is because the proposed optimal bundling treats both the gateway node and leaf node equally since they are most likely the same resource-constrained sensor nodes in the real deployment and the gateway nodes undertake more traffic than the leaf ones. However, when the gateway node is resource-abundant rather than resource-constrained, consequently the proposed approach should incline the optimization to the leaf nodes.

In addition, as briefly mentioned in Section 5.4, the maximum energy efficiency performance of the proposed optimal bundling is not exhibited due to the joint constraints of the network size and various parameters such as maximum bundling number. In the follow-up work, a further study will employ a relative larger testbed and proper parameters to evaluate the extreme performance of the proposed optimal bundling.

## 6. Conclusions

We have proposed an approach to optimize the number of bundled messages at sensor nodes in a WSN under the constraints of time synchronization accuracy and E2E delay. In order to solve the optimal bundling problem, we formulate it as an ILP model and employ the novel asymmetric time synchronization scheme. To the best of the authors’ knowledge, this is the first work to optimize message bundling for energy efficiency under the joint constraints of synchronization accuracy and E2E delay in the context of WSNs. The practical evaluation results on a real testbed composed of TelosB sensor nodes demonstrate the long-term and runtime maintenance capability of the proposed approach in terms of E2E delay. The energy efficiency is also illustrated through a case study, showing about 70% reduction on the overall message transmissions.

Note that bundling a larger number of messages results in a longer payload, which may lead to possible link degradation such as the packet reception ratio degradation. In this regard, link quality requirements could be introduced to the optimization model as additional constraints to take into account more impacts on the overall performance by the bundling procedure. In addition, the proposed approach should be tested with a larger testbed to investigate its scalability and network-wide energy efficiency.

## Figures and Tables

**Table 1:** MAE and MSE of measurement time estimation of EE-ASCFR and AHTS with different synchronization intervals (SIs) provided in [1] and [43].

Synchronization Scheme		MAE ^1^	MSE ^2^
EE-ASCFR	SI=100s	8.8811 × 10${}^{-25}$	5.8990× 10${}^{-19}$
	SI=1s	9.1748 × 10${}^{-25}$	5.4210 × 10${}^{-19}$
	SI=10ms	1.0887 × 10${}^{-24}$	4.7684 × 10${}^{-19}$
AHTS	SI=100s	8.4225 × 10${}^{-6}$	1.2524 × 10${}^{-10}$
	SI=10s	2.3385 × 10${}^{-6}$	9.1694 × 10${}^{-12}$
	SI=1s	1.8166 × 10${}^{-6}$	5.2094 × 10${}^{-12}$

${}^{1}$ MAE is the mean absolute error of measurement time estimation. ${}^{2}$ MSE is the mean square error of measurement time estimation.

**Table 2:** Optimal bundling number under different end-to-end (E2E) delay requirements during the dynamic experiment as shown in Figure 9b.

Max Bundling Number	E2E Delay Requirement (s)	Optimal Bundling Number			
		Node 1	Node 2	Node 3	Node 4
15	14	15	14	8	1
15	10	15	10	6	1
15	8	15	6	4	1
15	6	15	2	2	1
15	4	14	1	1	1
15	1	1	1	1	1
